# Genetic Model Identification and Major QTL Mapping for Petiole Thickness in Non-Heading Chinese Cabbage

**DOI:** 10.3390/ijms25020802

**Published:** 2024-01-09

**Authors:** Guangyuan Liu, Yongkuan Li, Jia Si, Rong Lu, Maixia Hui

**Affiliations:** Vegetables Engineering and Technology Research Center of Shaanxi Province, College of Horticulture, Northwest A & F University, Yangling, Xianyang 712100, China; liugy202308@nwafu.edu.cn (G.L.); liyongkuan@nwafu.edu.cn (Y.L.); sijia@nwafu.edu.cn (J.S.); lurong@nwafu.edu.cn (R.L.)

**Keywords:** non-heading Chinese cabbage, petiole thickness, genetic model, major QTL, *SMXL6*

## Abstract

Petioles of non-heading Chinese cabbage are not only an important edible part but also a conduit for nutrient transport, holding significant agricultural and research value. In this study, we conducted a comprehensive genetic analysis of petiole-related traits using a segregating population. Modern quantitative genetic approaches were applied to investigate the genetic regulation of petiole thickness. The results indicated that petiole thickness is a quantitative trait, and the identified genetic model was consistent with two pairs of additive-dominant main genes and additive-dominant polygenes (2MG-AD). BSA-seq analysis identified a major effect of QTL controlling petiole thickness on chromosome A09: 42.08–45.09 Mb, spanning 3.01 Mb, designated as *QTL-BrLH9*. Utilizing InDel markers, the interval was narrowed down to 51 kb, encompassing 14 genes with annotations for 10 of them. Within the interval, four mutated genes were detected. Combined with gene annotation, protein sequence analysis, and homology alignment, it was found that *BraA09g063520.3C*’s homologous gene *SMXL6* in Arabidopsis (*Arabidopsis thaliana* (L.) Heynh) is an inhibitor of the coding and synthesis of the strigolactone pathway. Strigolactone (*SLs*) plays an important role in plant growth and development. The cloning results showed that multiple frameshift mutations and non-synonymous mutations occurred on the exon. The qPCR results showed that the expression of the gene was significantly different between the two parents at the adult stage, so it was speculated that it would lead to changes in petiole thickness. *BraA09g063520.3C* was predicted as the final candidate gene.

## 1. Introduction

Non-heading Chinese cabbage (*Brassica campestris* ssp. *chinensis* Makino), which is a significant leafy vegetable native to China, has a wide cultivation area in the south and is widely planted in cities in the middle and lower reaches of the Yangtze River, accounting for 30–40% of the total vegetable output [1]. Its cultivation area increased from 533,000 hm^2^ in 2005 to about 1,333,300 hm^2^ in 2020, an increase of 2.5 times. It has played an important role in the annual production and supply of vegetables. Among them, the planting area of ordinary Chinese cabbage is 1,211,200 hm^2^, the amount of planting is 9084 tons, and the terminal output value is about 900 million yuan, with a huge growth space [2]. Each 100 g of non-heading Chinese cabbage contains 1.8 g of protein, 0.7 g of dietary fiber, 45 mg of vitamin C, 0.60 mg of vitamin A (which can be converted into vitamin A), 10.24 mg of vitamin E, and 262 mg of calcium. The four elements of calcium, magnesium, sodium, and potassium carry negative oxygen ions into the human body when they enter the body. It reaches various organs of the human body, neutralizes the acidic substances in the human body, decomposes the water molecules, and discharges them out of the body so that the acid and alkali of the human body are balanced, which plays an important role in human health. The petiole is rich in dietary fiber, which can increase gastrointestinal motility, help digestion, prevent constipation, and prevent gallstones, hyperlipidemia, cardiovascular disease, and diabetes [1].

Non-heading Chinese cabbage leaves consist of leaf blades and petioles, with the petiole serving as a transitional structure between the stem and leaf. It not only provides support for the leaf blade but also determines its positioning. Leaves originate from peripheral leaf primordial cells of the shoot apical meristem (SAM) and are characterized by the activation of leaf development-related genes. When leaf differentiation was initiated, the genes related to leaf primordium development began to express, while the SAM characteristic gene *knox1* family members did not express [3].

Plant hormones play a vital role in the initiation of leaf primordia. For instance, localized auxin concentration elevation triggers leaf primordium growth and differentiation. During early leaf primordium development, cells rapidly divide to increase their number. Subsequently, precise gene regulation guides polarized growth, leading cells toward specific directions for division and differentiation. This results in leaves with distinct morphology and structure [4].

Leaf development involves three axial dimensions: the proximal–distal axis (base–top axis) responsible for petiole and leaf differentiation, the medial–lateral axis along the midvein to leaf edge expansion, and the adaxial–abaxial axis determining leaf thickness [5]. In summary, polarity differentiation occurs along the adaxial–abaxial axis, proximal–distal axis, and medial–lateral axis [6]. The leaves are developed from the base–apical axis; the base axis develops into a petiole, and the apical axis develops into a leaf [7].

Six members of the yabby gene family involved in three axial directions in *A. thaliana*, including *AtCRABS CLAW*, *AtYABBY1*, *AtYABBY2*, *AtYABBY3*, *AtYABBY4*, and *AtYABBY5*, all encode transcription factors and are expressed in leaf primordia [8]. Mutants such as yab135 and yab1235 exhibit severe developmental defects, including a round, cylindrical leaf shape [9,10]. The *WOX* gene family, particularly *wox1* prs double mutants, affects the medial–lateral axis [11]. The *YUC* gene family, involved in auxin synthesis, influences medial–lateral axis development. AS1, AS2, HD-ZIPIII, ETT/ARF3, ARF4, KANADI, and YABBY transcription factors influence the adaxial–abaxial axis [11] and contribute to leaf adaxial and abaxial side development [12,13].

In maize (*Zea mays* L.), the Liguleless-narrow-reference (*Lgn-R*) gene mutation leads to reduced leaf width and length, indicating its involvement in both proximal–distal and medial–lateral axis development [14,15]. In *A. thaliana*, SLs (strigolactones) have emerged as regulators of various developmental aspects, including branching [16,17], root system architecture [18,19], leaf senescence [20,21], and secondary growth [22,23,24]. SL biosynthesis involves DWARF27, CCD7/MAX3, and CCD8/MAX4 enzymes. The MAX1 cytochrome P450 catalyzes the conversion of carlactone to carlactonic acid [25], a precursor to various SLs [26].

In the analysis of the influence of the main agronomic traits of non-heading Chinese cabbage on its yield per plant by the grey correlation method, it was found that petiole thickness had a significant impact on yield. Yu Zhaojun et al. found that there was a very significant positive correlation between yield per plant and petiole thickness of non-heading Chinese cabbage [27]. However, at present, there are few QTL mappings on petiole thickness, and there are few direct experiences that can be used for reference. However, in view of the important contribution of petiole thickness to yield, this study separated the plants with extreme petiole traits from the recombinant inbred lines (RIL) of non-heading Chinese cabbage materials ZY and XS as parents to construct genetic populations, respectively. The genetic law of petiole-related traits was explored by phenotypic identification of petiole-related traits, and recombinant plants were screened by developing molecular markers to locate candidate genes for petiole-related traits. The results of this study are of great significance to the study of the genetic law of petiole-related traits, the molecular-assisted selection breeding of non-heading Chinese cabbage, and the theoretical study of breeding high-yield and high-quality varieties and related genes. At the same time, it also enriches the genetic law and genes of non-heading Chinese cabbage petiole.

## 2. Results

### 2.1. Petiole Thickness Phenotypic Identification and Genetic Analysis

**Figure 1:**
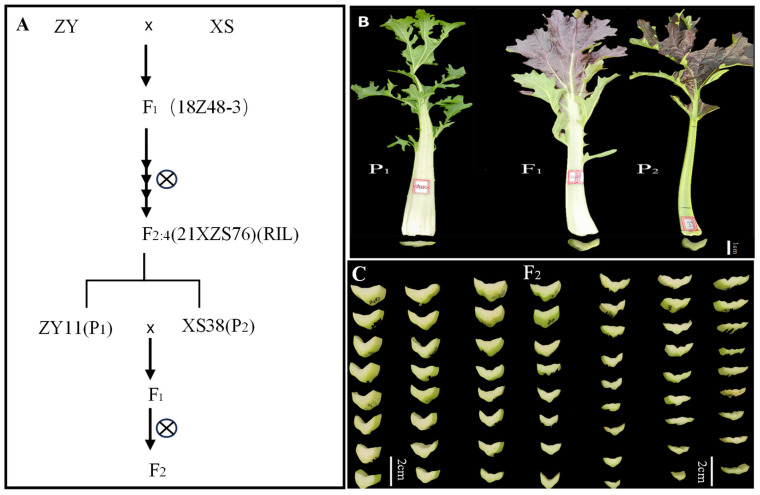
Segregation population construction process and petiole phenotype. (**A**) The extreme individuals from F_2:4_ (RIL) family (21XZS76) were selected as parents to construct F_1_ and segregate the population. (**B**) The petiole phenotype of parents and F_1_ phenotype, P_1_ (ZY11) petiole thin, P_2_ (XS38) petiole thick, F_1_ petiole thick. (**C**) Part of the F_2_ plant petiole thickness changes.

**Figure 2:**
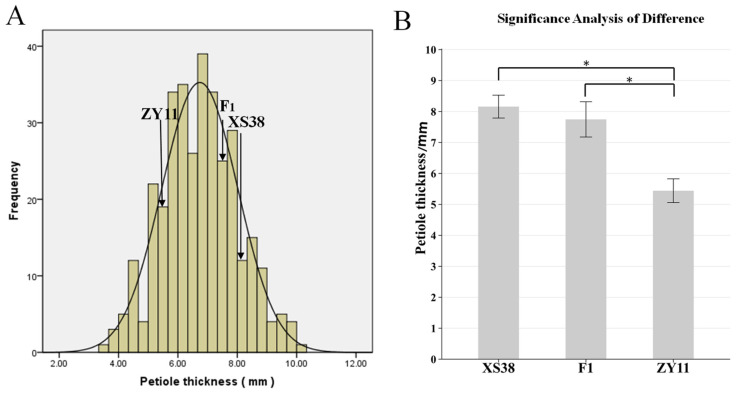
F_2_ petiole thickness frequency distribution histogram and parental petiole thickness difference significance analysis. (**A**) F_2_ mapping population and the distribution of petiole thickness at different times. (**B**) Significant difference analysis between XS38 and ZY11 and F_1_, * *p* < 0.05.

The petiole of parent XS38 was thicker; the petiole of parent ZY11 was thinner (Figure 1B); and the petiole thickness of F_1_ was closer to that of parent XS38. The petiole thickness of F_2_ individuals gradually decreased from left to right, and the change process was obvious (Figure 1C). The petiole thickness of F_2_ was measured and plotted. It was found that the distribution was continuous and normal. The normality test of petiole thickness was analyzed by IBM SPSS Statistics 19 software. The skewness = 0.101, the kurtosis = −0.23, and the absolute values of skewness and kurtosis were in the range of 0–1. It can be considered that the kurtosis is a typical normal peak. According to this, petiole thickness is a quantitative trait. At the same time, according to the difference significance analysis of the petiole thickness of the parents ZY11 and XS38, it is found that it is within the 95% confidence interval. The thickness difference between the two was extremely significant. The petiole thickness of the hybrid F_1_ and the thickness of ZY11 were extremely significant (Figure 2). At the same time, it was found that the petiole thickness of F_1_ was closer to that of XS38, showing a thicker petiole thickness (Table 1).

Genetic models were identified and analyzed based on petiole thickness data. The genetic regulation of petiole thickness was investigated. The optimal genetic model controlling petiole thickness was determined to be 2MG-AD, with an AIC value of 1132.9. In this model, the petiole thickness trait was regulated by two pairs of additive-dominant major genes and additive-dominant polygenes. The parameters of this model were: The first mean of the population was m1 = 8.9251, m2 = 7.97, and m3 = 7.14; the corresponding additive variance was 0.4483; the overall population mean was m = 6.6989; the additive effect value of the first major gene was da = 1.3361; and the heritability of the major gene was 72.72%. The optimal genetic model was marked in deep blue according to AIC criteria, while potential genetic models were marked in light blue. However, all of these models exhibited a situation controlled by major genes (Table 2). Considering the results of this analysis and the fitness test parameters, obtaining QTLs through sequencing was deemed feasible. Theoretical distribution curves based on the genetic model were plotted (Supplementary Figure S1), showing a consistent trend with the previously conducted normality test, thereby reinforcing mutual validation.

**Figure 3:**
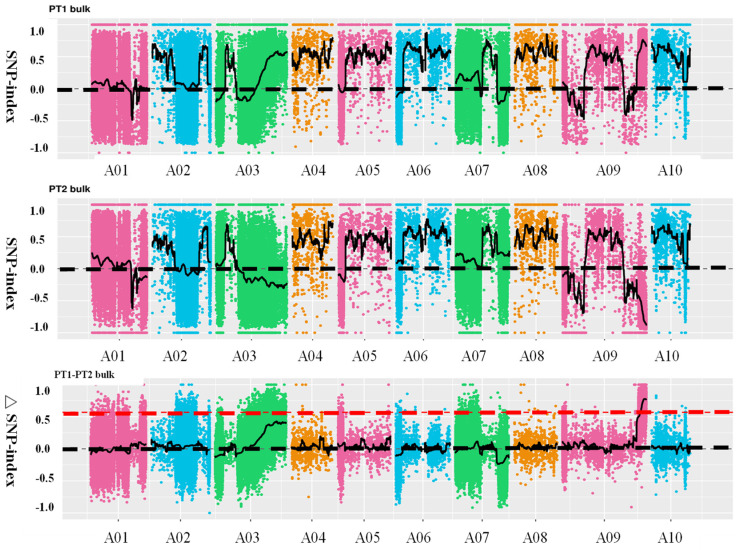
Distribution of SNP (single nucleotide polymorphism)-index correlation values along chromosomes. The abscissa represents chromosome names, colored dots represent calculated SNP-index (or ΔSNP-index) values, and Black dotted line is coordinate line, black solid line represent fitted SNP-index (or ΔSNP-index) values. The upper panel depicts the distribution of SNP-index values for the PT1 (thick petiole 1-dominant) bulk; the middle panel illustrates the distribution of SNP-index values for the PT2 (thin petiole 2-recessive) bulk; the lower panel shows the distribution of ΔSNP-index values, with the red line indicating the threshold line at the 99th percentile.

Using SNP (single nucleotide polymorphism) values from the extreme pools within the F_2_ population, the SNP-index was calculated, and the difference in SNP-index values between the two pools, denoted as Δ(SNP-index), was plotted against chromosome positions. The intervals with relatively large Δ(SNP-index) values were considered candidate regions for the target trait. Ultimately, one candidate region was identified on chromosome A09, spanning 42.08–45.09 Mb, with a total length of 3.01 Mb (Figure 3). This region was named the principal *QTL-BrLH9*. Integrating chromosome distribution, gene distribution, SNP distribution, ED association values, and Δ(SNP-index) distribution into a comprehensive graphical representation, the results were visualized using Circos (http://circos.ca/) software (Supplementary Figure S2).

### 2.2. Fine Mapping of Principal QTL BrLH9 for Petiole Thickness

**Figure 4:**
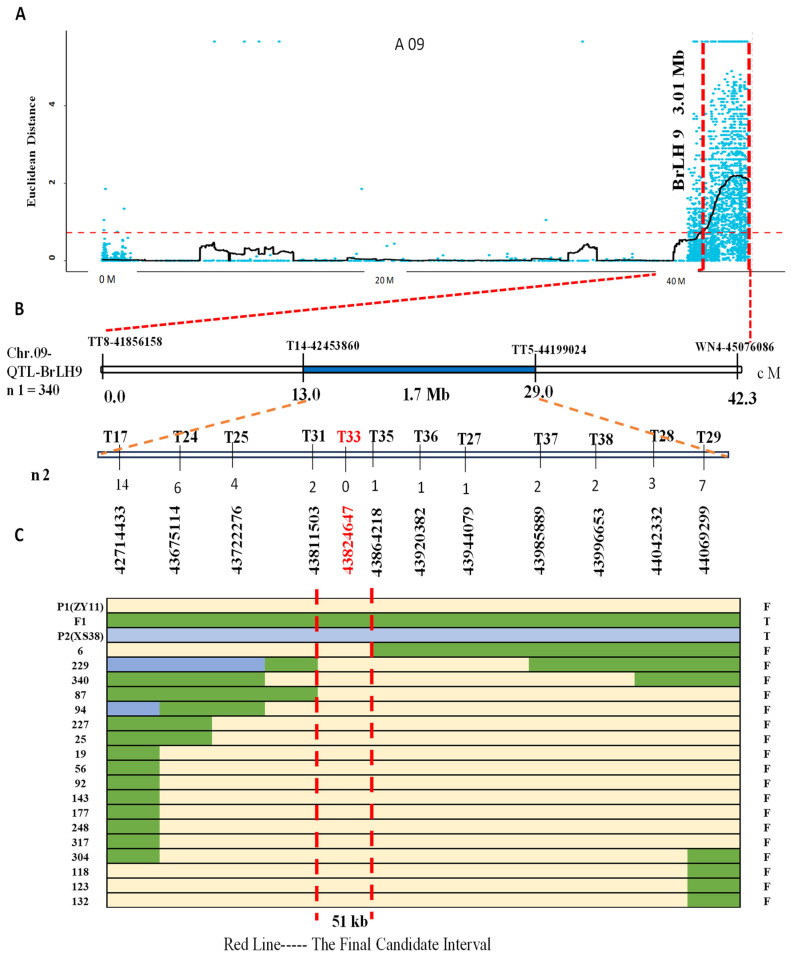
Fine mapping of main effect *QTL-BrLH9*. (**A**) SNP candidate regions associated with the ED (Euclidean distance) algorithm on chromosome A09. Blue dots represent calculated ΔSNP-index values, with the main effect of *QTL-BrLH9* covering a region of 3.01 Mb indicated by the red horizontal line, and the vertical red line representing the associated region. (**B**) Primer sets for fine mapping within the interval, where the first four represent initial dual-flanking primers. The primer names and positions are shown at the top, and genetic distances and interval sizes are provided at the bottom, spanning 1.7 Mb. The chromosome location is indicated on the left. The F_2_ mapping population used comprises n1 = 340, with n2 denoting the number of recombinant individuals exhibiting the thin stem phenotype. Encrypted primer names are at the top, accompanied by the number of recombinants and physical positions. Notably, no recombinants were identified by Marker T33, The blue area is the initial positioning interval. (**C**) Different colored lines represent different genotypes: Yellow for P_1_ (ZY11), green for F_1_, and light blue for P_2_ (XS38). The left end of each line indicates the identifier of parental, F_1_, and F_2_ recombinant individuals, while the right end indicates the stem phenotype: F for thin and T for thick. The red vertical line marks the final fine mapping interval of 51 kb.

Based on the sequencing results of PT1, PT2, and the parent lines, 38 pairs of InDel markers were designed. Among these, 16 pairs displayed polymorphism after validation by the parents (Table S1). Four markers, namely TT8, T14, TT5, and WN4, were initially employed to screen 340 F_2_ individuals; a total of 53 recombinant plants were screened out. Recombinant individuals were identified using these markers, further narrowing down the BrLH9 interval between molecular markers T14 and TT5 within a 1.7 Mb physical distance. For faster gene localization, lateral markers T14 and TT5 were utilized to screen 93 F_2_ individuals with a thin petiole phenotype, yielding 18 recombinants. Subsequent denser marker screening around Marker T33 revealed a reduction in recombinants from 14 to 0 on the left side and from 7 to 0 on the right side of Marker T33. The nearest markers to Marker T33 were Marker T31 and Marker T35. Accordingly, the interval was narrowed down to Marker T31~Marker T35, covering a physical distance of 51 kb (Figure 4). Marker T33 exhibited consistent band patterns among all recombinants within this interval. Primer Marker T33 was designed for the mutated InDel site of *BraA09g063520.3C*. The verification results of Marker T33 on all thin petiole phenotype plants showed the same band type as ZY11.

### 2.3. Prediction of Candidate Genes, Gene Expression Analysis and Gene Cloning Analysis

**Figure 5:**
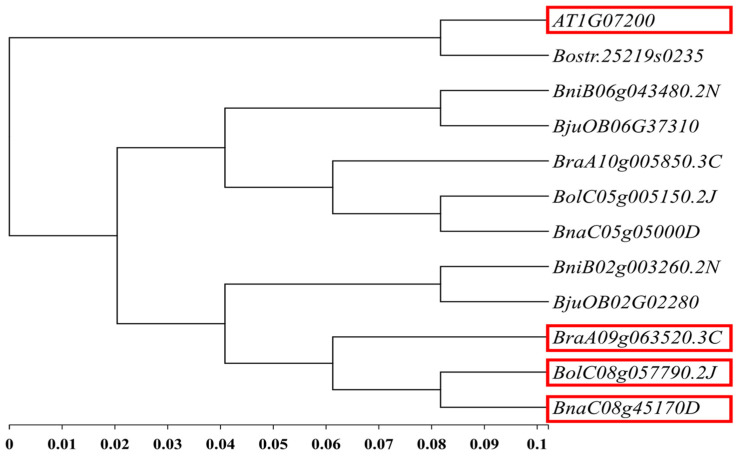
Phylogenetic analysis of candidate genes. The red box represents the homologous gene of *BraA09g063520.3C* in *B.oleracea*, *B.napus* and *A.thaliana*, and its functional annotation is SMXL6.

Based on the 51 kb interval of the main effect *QTL-BrLH9* and functional annotations from the *Brassica* database (BRAD), ten genes were annotated within the candidate region. These genes were aligned to *A. thaliana* information resources in the *Arabidopsis Information* Resource (TAIR) to search for homologous genes. According to BSA-seq data, three genes in this interval harbored non-synonymous mutations: *BraA09g063490.3C*, *BraA09g063520.3C*, and *BraA09g063530.3C*. Among them, *BraA09g063520.3C* also contained a frameshift mutation (Table 3). The *A. thaliana* homolog of *BraA09g063520.3C* was *AT1G07200*, also known as SMAX1-LIKE 6 (SMXL6). This gene was the closest to *BraA09g063520.3C* in rapeseed (*Brassica napus* L.), where it was annotated as SMAX1-LIKE 6 (Figure 5). SMXL6 is an inhibitor in the Strigolactone signaling pathway and exerts dual functions in protein–DNA binding. It represents the only gene within this interval that is clearly associated with the petiole phenotype. 

**Figure 6:**
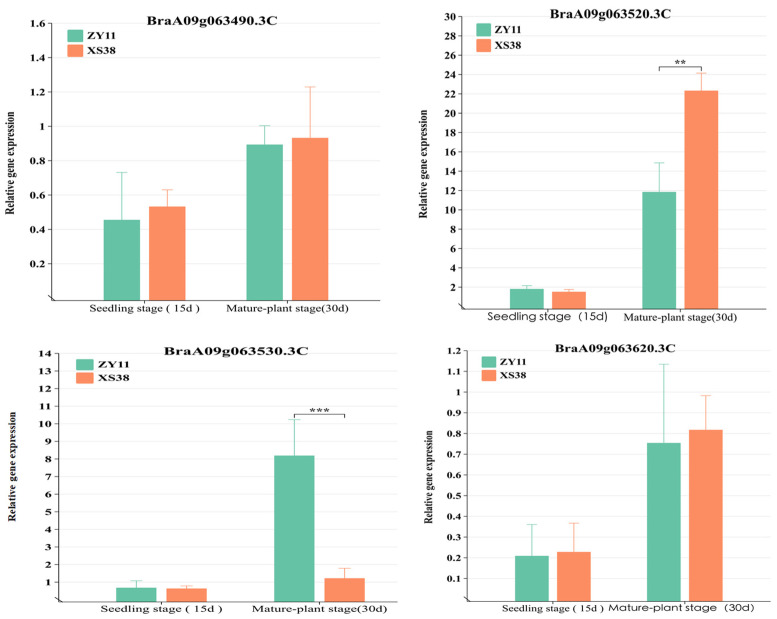
Analysis of the relative expression of candidate genes. The expression of *BraA09g063520.3C* at seedling stage. The expression of *BraA09g063520.3C* in the petiole tissue of the parent at seedling stage was not significantly different. The expression level of *BraA09g063520.3C* at adult stage was significantly higher in the thick petiole parent (XS38) than in the thin petiole parent (ZY11), ** *p* < 0.01, *** *p* < 0.001.

Four genes had mutations in exons. Specifically, *BraA09g063520.3C* displayed both frameshift and non-synonymous mutations. The relative expression levels of all genes in the interval were quantified in real-time fluorescent assays. The results showed differential expression of *BraA09g063520.3C* in the petiole tissue of parent materials. During the seedling stage (15 d), the difference in expression between thick-petioled ZY11 and thin-petioled XS38 was not significant and was low. This might be attributed to the early initiation of expression and the limited phenotypic differences at that stage. However, during the mature stage (30 d), expression of *BraA09g063520.3C* in the thick-petioled XS38 was significantly higher than in the thin-petioled ZY11, reflecting the differences in petiole thickness between the two types (Figure 6). For *BraA09g063530.3C*, there was no significant expression difference in the seedling stage’s petioles (15 d), while in mature plants, it was significantly more expressed in ZY11 than in XS38. Expression of the other mutated genes within the interval showed low and non-significant differences in expression between the parent materials. The relative expression levels of other genes in the interval were low and exhibited insignificant differences. 

**Figure 7:**
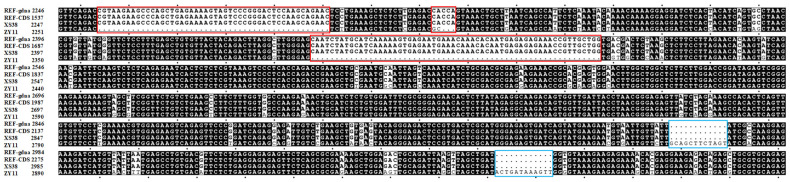
The cloning results of *BraA09g063520.3C* in ZY11 and XS38 (REF-gdna, REF-CDS, XS38, and ZY11 sequences from top to bottom on the left). Compared with the full-length genome and CDS (coding sequence) of the reference genome, no frameshift mutation was found in XS38. There were 5 frameshift mutations in the exon of ZY11, and the frameshift mutation at the blue frame line was the frameshift mutation on Marker T33. The black background is the common sequence of the four, the white background is the difference base, and the red frame line is the frameshift mutation.

Furthermore, the full-length genome of the gene *BraA09g063520.3C* in the parents ZY11 and XS38 was compared with the software SNAPGENE (www.snapgene.com). The total length of the reference genome was 3565 bp, the ZY11 clone sequence was 3483 bp, and the XS38 was 3565 bp. The results showed that the sequence of XS38 had individual base mutations, and the frameshift mutation length was equal to the reference genome sequence. In ZY11, there were multiple frameshift mutations and non-synonymous mutations. There were two frameshift mutations in the amplification length range of Marker T33, resulting in the deletion of large fragments (Figure 7), which also explained that Marker T33 was completely consistent in the vertical gel electrophoresis of thin petioles and could well distinguish thin and thick petioles through this marker. The cloning results showed that there were 5 frameshift mutations and 2 frameshift mutations on primer T33.

## 3. Discussion

### 3.1. Analysis of Petiole Thickness Genetic Regulation

Combining parent and F_1_ petiole thickness phenotypic data, it was observed that F_1_ petiole thickness closely resembled that of Parent 2 (XS38). The petiole thickness displayed a typical normal distribution with continuous variation, indicating a quantitative trait. Applying modern genetic analysis methods, the optimal genetic model controlling petiole thickness was identified as 2MG-AD, involving two pairs of additive-dominant major genes and additive-dominant polygenes. Under this model, the first mean of the F_2_ population was 8.9251, with a component distribution variance of 0.4483. This implies the average petiole thickness in the F_2_ population and the extent of variation explained by genetic factors. The overall population mean was m = 6.6989, representing baseline petiole thickness. The additive effect value of the first major gene was da = 1.3361, indicating the contribution of this gene to the increase in petiole thickness. The additive variance of the F_2_ major gene was 1.1951, providing insight into the genetic variability attributed to this specific major gene. The heritability of the major gene was 72.7209%, suggesting a high degree of genetic control over petiole thickness. Prior research on petiole weight suggested a genetic model controlled by a pair of additive major genes and memory-dominant polygenes [28]. Similarly, investigations on other *Brassica*, such as cabbage (*Brassica oleracea* L. var. *Capitata* Alef. f. *alba* DC.), implied a genetic model involving two pairs of major genes and polygenes for leaf length and width [29]. In non-heading Chinese cabbage, genetic models controlling traits including plant height, bolting degree, maximum leaf length, maximum leaf width, and leaf color were also identified to be regulated by two pairs of major genes and polygenes [30]. However, limited research has been conducted on the genetic analysis of petiole thickness and leaf thickness, possibly due to the labor-intensive nature of measurements. Hence, this study focused on the genetic analysis of petiole thickness in non-heading Chinese cabbage, which is controlled by two pairs of major genes and additive-dominant polygenes. The integration of this analysis with BSA-seq facilitated the identification of the main effect of QTL on petiole thickness, providing a solid foundation for future gene cloning and molecular marker development.

### 3.2. Fine Mapping of Main Effect QTL for Petiole Thickness and Prediction of Candidate Genes

By constructing a recombinant inbred line (RIL) population and employing BSA-seq, the principal QTL for non-heading Chinese cabbage petiole thickness, *BrLH9*, was mapped onto chromosome A09, spanning the physical position 42.08–45.09 Mb with a total length of 3.01 Mb. Through the integration of parent data and sequencing of extreme individual pools, the interval was further narrowed down to Marker T14-Marker TT5, covering a size of 1.7 Mb. Subsequent denser marker screening led to the precise localization of the candidate region between Marker T31 and Marker T35, spanning 51 kb. Validation of Marker T33 on all thin petiole phenotype plants confirmed identical banding patterns with ZY11, suggesting that Marker T33 was linked to the gene associated with the thin petiole phenotype. Its function is annotated as SMAX1-LIKE 6. Strikingly, SMAX1-LIKE 6 (SMXL6) and its homologs in *A. thaliana* and rice (*Oryza sativa* L.), SMAX1 and DWARF53, are downstream targets of MAX2 in strigolactone signaling. *SMXL6/7/8* triple mutants exhibit inhibited stem elongation, accompanied by reduced leaf width and length [31]. SMXL6 accumulation promotes shoot branching and leaf elongation in *A. thaliana*, demonstrating a new function in branching and leaf development regulation. The accumulation of SMXL6 upregulates *BRC1* expression, disrupting hormone balance to regulate branching. SMXL6 is also involved in auxin and cytokinin signal pathways by regulating *TCP1* expression, thereby influencing leaf shape and petiole length [32]. In conclusion, *BraA09g063520.3C*, the candidate gene, is a homolog of SMXL6 in *A. thaliana*, acting as a transcriptional repressor in the strigolactone pathway, with a dual DNA-binding function [33]. 

### 3.3. Relative Expression and Cloning Analysis of Candidate Genes

Among the 10 annotated genes within this region, *UGT71B6*, a homologous gene of *BraA09g063490.3C* in *A. thaliana*, may be involved in plant stress responses mediated by the ABA receptor [34]. *BraA09g063490.3C* contains an unknown functional domain of DUF, which has been speculated to be involved in the stress process of plants [35]. *BraA09g063520.3C* is annotated as SMAX1-LIKE 6 (SMXL6) protein. SMXL6 acts as an inhibitor to regulate leaf morphology and plant branching in *A. thaliana*. Researchers have carried out extensive research on strigolactone response genes, but so far only a few have been identified, including *SMXL2*,*6*,*7*,*8* in *A. thaliana*. Strigolactone promotes leaf elongation in an EAR-dependent manner through SMXL6, increasing the possibility that strigolactone also regulates leaf development through other downstream genes [36]. *BraA09g063530.3C* homologous gene SIRANBP in *A. thaliana* is involved in regulating stomatal opening and closing, thus participating in plant disease resistance regulation [37]. *BraA09g063550.3C* contains a highly conserved domain that catalyzes protein activity [38]. In *A. thaliana*, the homologous gene family *LSH* of *BraA09g063560.3C* is involved in the development of lateral organs and forms additional buds or bud organs in flowers during the reproductive period [39]. *BraA09g063570.3C* belongs to the thioredoxin superfamily protein and is involved in the regulation of resistance to downy mildew [40]. *BraA09g063580.3C* is involved in the resistance to clubroot in Chinese cabbage (*Brassica rapa* L. var. *glabra* Regel) [41]. *BraA09g063600.3C* is identified as a salt tolerance-related gene in *A. thaliana* [42]. *BraA09g063620.3C* is involved in abiotic stress [43], and the functions of other genes are unknown. 

In natural populations, functional loss variants (premature stop codons, frameshift mutations, splice site alterations, and start codon losses) result in gene inactivation and are considered natural mutants [44]. *BraA09g063520.3C* is the only double mutant gene in the interval, and its homologous gene is *SMXL6*. SMXL6, SMXL7, and SMXL8 play a regulatory role in drought response. The double and triple mutants found that the *SMXL6*/*7* double mutant and the *SMXL6*/*7*/*8* triple mutant showed longer cotyledon petioles, and the *SMXL6*/*7*/*8* triple mutant showed longer and narrower leaves than WT, and its survival rate under drought conditions was significantly higher than that of wild-type WT. Using projection electron microscopy to determine the cuticle thickness of wild-type and double and triple mutants, it was found that the thickness of double and triple mutants was significantly thicker than that of wild-type. As important inhibitors of MAX2 in strigolactone signal transduction, SMXL6 (SUPPRESSOR OF MAX2-LIKE6), SMXL7, and SMXL8 are involved in plant growth and development regulation and salt stress response. Through SEM observation, it was found that the length-width ratio of leaf epidermal cells of the *SMXL6/7/8* triple mutant increased, the cell area decreased, and the number of cells per unit area increased. The results of the transcriptome showed that the expression patterns of cytokinin, auxin, gibberellin signal, and other related genes in the *SMXL6*/*7*/*8* triple mutant were changed compared with wild type, indicating that D53-like SMXLs may regulate the development of leaf morphology by affecting the expression of these signal-related functional genes [45]. Furthermore, SMXL6, SMXL7, and SMXL8 play roles in drought response and salt stress adaptation. SMXL2, as a negative regulator of strigolactone signaling, also affects plant growth and drought resistance. SMXL6 degradation induces the expression of *BRC1, TCP1*, and *PAP1*, promoting stem branching inhibition, enhancing leaf elongation, and activating anthocyanin biosynthesis [46]. There are five frameshift mutations in the exon of ZY11, which lead to changes in the protein sequence and also have an important impact on the function of the gene, laying a solid foundation for the follow-up study of the gene. Combined with the above research results, it is speculated that this gene plays a positive regulatory role in petiole thickness. Hence, *BraA09g063520.3C* was predicted as the candidate gene.

## 4. Materials and Methods

### 4.1. Plant Materials and RIL Population Construction and Phenotypic Identification

The materials used in this experiment were all owned by the Cruciferous Vegetable Crop Genetic Improvement Technology Innovation Laboratory of Northwest A&F University. The F_1_ generation was obtained through the crossbreeding of non-heading Chinese cabbage ZY and XS, with continuous self-pollination to produce the inbred line F_2:4_ (RIL). Among them, ZY11 with extremely thin petiole was selected from F_2,4_ family 21XZS76 as parent P_1_, XS38 with extremely thick petiole was selected as parent P_2_, and F_1_ was obtained by hybridization. F_1_ was self-pollinated to obtain the F_2_ segregating population (Figure 1A). The above non-heading Chinese cabbage RIL population, parents ZY11 and XS38, were planted in the greenhouse of Caoxinzhuang Experimental Farm at Northwest A&F University, and routine management was carried out. Generation groups P_1_, P_2_, F_1_, and F_2_ were planted in the Caoxinzhuang experimental farm. The inbred lines, parents, and F_1_ generations of the RIL population were planted with 20 plants each, three blocks each, and 360 plants were planted in the F_2_ generation. The plant spacing was 20 cm, and the row spacing was 25 cm. After 30–40 days of planting, 

According to the quality control standard of non-heading Chinese cabbage germplasm resources [47] (https://www.cgris.net) and the method in the article [48,49]: Measure the thickness of petiole; in the normal harvest period, a complete plant has about 15–19 leaves, every 3–4 leaves form a round, and the largest rosette leaf is generally the second round of leaves from the outer wheel, that is, the secondary wheel. This round also has about 3–4 leaves, and the largest rosette leaf is also in this layer, that is, the fourth to eighth leaves from the outer wheel to the secondary wheel are the largest rosette leaves. When measuring the thickness of the petiole, the thickness of the thickest petiole of the largest rosette leaf in this period is selected as the thickness of the petiole, and the unit is mm for a total of 4 times. SPSS was used to analyze the difference significance and normality test of 45 strains of P_1_, P_2_, F_1_, and 340 strains of F_2_. The main gene + polygene mixed analysis of petiole thickness was performed using the R software package SEAv2.0. The genetic law of petiole thickness was analyzed [50].

### 4.2. BSA-Seq Method for Gene Pool Sequencing and Analysis

Genomic DNA was extracted using a modified CTAB method. Twenty individuals with consistent phenotypes from each parent line (XS38 and ZY11) were selected, and their DNA was pooled for parent pools. Thirty plants, each with extremely thin and thick petioles, were selected from the F_2_ segregation population, and their DNA was pooled to create the F_2_ thick petiole pool (PT1) and the F_2_ thin petiole pool (PT2). The four DNA pools were sequenced by Beijing Biomarker Technologies Co., Ltd. (Illumina, San Diego, CA, USA). Sequencing depths were 20× for parent pools and 30× for F_2_ pools. The *Brassica rapa* chiifu V3.0 reference genome was used. SNP detection was performed using the Haplotype Caller algorithm of GATK (Genome Analysis Toolkit) for SNP and InDel filtering. High-quality SNP and InDel sites were obtained through consistent filtering criteria, and candidate regions associated with SNPs were analyzed.

### 4.3. Development of Molecular Markers

Based on the sequencing data of parent lines and the F_2_ population, Indels were selected for marker development, prioritizing those with high sequencing depth and fragment differences greater than 5 bp. Primers were designed using Primer Premier 5.0 software and synthesized by Shanghai Sangon Biotech Co., Ltd. (Shanghai China). Genotyping of PCR products was conducted using 8% polyacrylamide gel electrophoresis. Bands consistent with ZY11 were labeled as “A”; those consistent with XS38 were labeled as “B”; and hybrids were labeled as “H” [51].

### 4.4. Candidate Gene Identification

Using the *Brassica rapa* chiifu V3.0 reference genome, genes within candidate regions were annotated. Mutations in candidate genes were analyzed based on parent and F_2_ sequencing data, with a specific focus on frameshift and non-synonymous mutations. Preliminary candidate gene identification was aided by homologous gene function analysis through gene cloning.

### 4.5. Candidate Gene Identification, Relative Expression and Gene Cloning

The genes in the candidate interval were annotated according to the reference genome, *Brassica rapa* chiifu V3.0. Combined with the parents and F_2_ sequencing data, the mutation sites of the candidate genes were analyzed, and the frameshift mutation genes and non-synonymous mutation genes were analyzed. Through the functional analysis of homologous genes in gene cloning, the candidate genes were preliminarily determined. For the four genes mutated in the candidate interval, qPCR was performed to measure the relative expression of the genes in the petiole tissues at the seedling stage (15 d) with insignificant petiole differentiation and the adult stage (30 d) with obvious petiole differentiation. Due to the extended length of the candidate gene, primers were designed to clone the full length of the genome. After cloning, the product was connected to the vector and then transferred to *E. coli* to select a single colony. The colony was sent to the Beijing Aoke Biological Company for sequencing and sequence alignment analysis.

## 5. Conclusions

Petiole thickness is regulated by two pairs of additive dominant major genes + additive dominant polygenes, which shows dominance for petiole thinness. The main QTL for petiole thickness, *BrLH9*, was identified by the BSA-seq method. The physical location is in the 42.08–45.09 Mb genomic region on chromosome A09. Combined with the recombinant plant, the interval was reduced to 51 kb, and there were four mutant genes in the interval. According to the difference analysis of relative expression in parents and the results of gene cloning, the candidate gene was speculated to be *BraA09g063520.3C*, which provided a theoretical basis and genetic resources for further enriching the genetic law of petiole thickness and related genes.

## Figures and Tables

**Table 1:** Analysis of variance and significance analysis between XS38 and ZY11 and F_1_.

Number of Generations	Number of Observations	Sum	Average	Standard Deviation	Variance	Source of Variance	SS	df	MS	F	*p*-Value	F Crit
XS38	45	367.1555	8.159011	0.3699	0.136825	Groups	192.9549	2	96.47747	475.3145	4.81 × 10^−61^	3.064761
ZY11	45	244.89	5.442	0.3837	0.147216	Interclass	26.79284	132	0.202976			
F1	45	348.6	7.746667	0.5700	0.324886	Grand Total	219.7478	134				

**Table 2:** Petiole thickness major gene + polygene mixed genetic analysis.

Model	Log_Max_ Likelihood_ Value	AIC ^a^	Mean (1) ^g^	Mean (2)	Mean (3)	Var (Residual + Polygene)	m ^f^	da(d) ^b^	Major-Gene Var ^c^	Heritabiliy (Major-Gene) (%) ^d^	P (U1 Square) ^e^	P (U2 Square)	P (U3 Square)	P (nW Square)	P (Dn)
0MG	−566.397	1136.794	6.7361			1.6435					0.8136	0.8378	0.9248	0.9262	0.8075
1MG-AD	−565.1425	1138.285	8.1174	6.7038	5.4236	0.9006	6.7705	1.3469	0.7429	45.202	0.9958	0.9918	0.9837	0.9838	0.9419
1MG-A	−565.1938	1136.388	8.0648	6.7365	5.4081	0.9734	6.7365	1.3284	0.67	40.7693	0.9831	0.9861	0.9901	0.9847	0.9304
1MG-EAD	−565.6526	1139.305	7.0077	5.9233		1.5745	6.4655	0.5422	0.0689	4.1941	0.9823	0.9717	0.9553	0.9871	0.9824
1MG-NCD	−565.5286	1139.057	6.3538	7.8788		1.2018	7.1163	0.7625	0.4417	26.8738	0.9699	0.9594	0.9544	0.9865	0.984
2MG-ADI	−565.4898	1150.98	7.9624	7.9612	7.7461	1.1216	6.9608	0.8935	0.5219	31.7534	0.9783	0.9847	0.9774	0.986	0.9892
2MG-AD *	−560.476	1132.952	8.9251	7.9716	7.1449	0.4483	6.6989	1.3361	1.1951	72.7209	0.9801	0.9687	0.9518	0.9999	0.9999
2MG-A	−566.2147	1140.429	7.0582	6.9533	6.8484	1.8461	6.7395	0.201	0	0	0.885	0.9264	0.8487	0.9624	0.8491
2MG-EA	−564.6005	1135.201	8.9814	7.8653	6.7492	0.4966	6.7492	1.1161	1.1469	69.7841	0.9882	0.9597	0.7952	0.9832	0.9703
2MG-CD	−566.4023	1140.805	6.8395	6.6503	6.6151	1.6225	6.6327	0.1122	0.021	1.2773	0.8121	0.8429	0.8982	0.9245	0.7983
2MG-EAD	−566.4024	1138.805	6.8408	6.6314	6.422	1.6222	6.6314	0.1047	0.0213	1.2949	0.8121	0.843	0.8978	0.9246	0.8

* Based on the predicted genetic model, dark green represents the optimal genetic model, and light green represents the potential genetic model. ^a^ AIC (Akaike’s information criterion), select the AIC value phase. The potential optimal inheritance of the quantitative trait is for several small genetic models. ^b^ da(d)b (additive effect of major gene). ^c^ Genetic variance of major genes. ^d^ major gene heritability. ^e^ U1-U3 (homogeneity test), nW (Smirnov test), Dn (Kolmogorov test). The three are collectively referred to as suitability test. ^f^ m (population means). ^g^ mean (1)–mean (3). The first, second, and third mean of a group.

**Table 3:** Candidate interval gene function annotation and variation information.

Gene ID	Homologous Genes in *A. thaliana*	The Name of *A. Thaliana* Gene	Number of Frameshift Mutations and Non-Synonymous Mutations	Functional Annotation
*BraA09g063490.3C*	*AT1G07250*	*UGT71C4*	0/11	Flavonol 3-O-glucosyltransferase
*BraA09g063500.3C*	*AT1G07220*	*-*	0/0	O-glucosyltransferase rumi homolog
*BraA09g063510.3C*	*AT1G07210*	*RIBOSOMAL PROTEIN BS18M*	0/0	Uncharacterized protein
*BraA09g063520.3C*	*AT1G07200*	*SMXL6*	6/20	SMAX1-LIKE 6
*BraA09g063530.3C*	*AT1G07140*	*SIRANBP*	0/4	Intracellular transport
*BraA09g063540.3C*	*-*	*-*	0/0	Uncharacterized protein
*BraA09g063550.3C*	*AT1G07110*	*FRUCTOSE-2,6-BISPHOSPHATASE*	0/0	6-phosphofructo-2-kinase/Fructose-2,6-bisphosphatase
*BraA09g063560.3C*	*AT1G07090*	*SITIVE HYPOCOTYLS-6; LSH6*	0/0	Protein LIGHT-DEPENDENT SHORT HYPOCOTYLS 6
*BraA09g063570.3C*	*AT1G07080*	*-*	0/0	Gamma-interferon-responsive lysosomal thiol protein
*BraA09g063580.3C*	*AT1G06990*	*-*	0/0	GDSL esterase/lipase *AT1G06990* isoform X1
*BraA09g063590.3C*	*AT1G06980*	*-*	0/0	Domain of unknown function
*BraA09g063600.3C*	*AT1G06970*	*ATCHX-14*	0/0	PREDICTED: LOW QUALITY PROTEIN: cation/H(+) antiporter 14
*BraA09g063610.3C*	*AT1G06930*	*-*	0/0	Uncharacterized protein
*BraA09g063620.3C*	*AT1G06923*	*-*	0/1	Transcription repressor *OFP17*

## Data Availability

Data is contained within the article.

## Supplementary Materials

The supporting information for this study can be downloaded at: https://www.mdpi.com/article/10.3390/ijms25020802/s1.
