# Supplementary material for: Genetic Model Identification and Major QTL Mapping for Petiole Thickness in Non-Heading Chinese Cabbage
Source: Int J Mol Sci. 2024 Jan 9;25(2):802. doi: 10.3390/ijms25020802 (PMC10815893; doi:10.3390/ijms25020802)
Supplement: Supplementary file 1 [file ijms-25-00802-s001.zip › ijms-2785048-supplementary.pptx]

## Slide 1
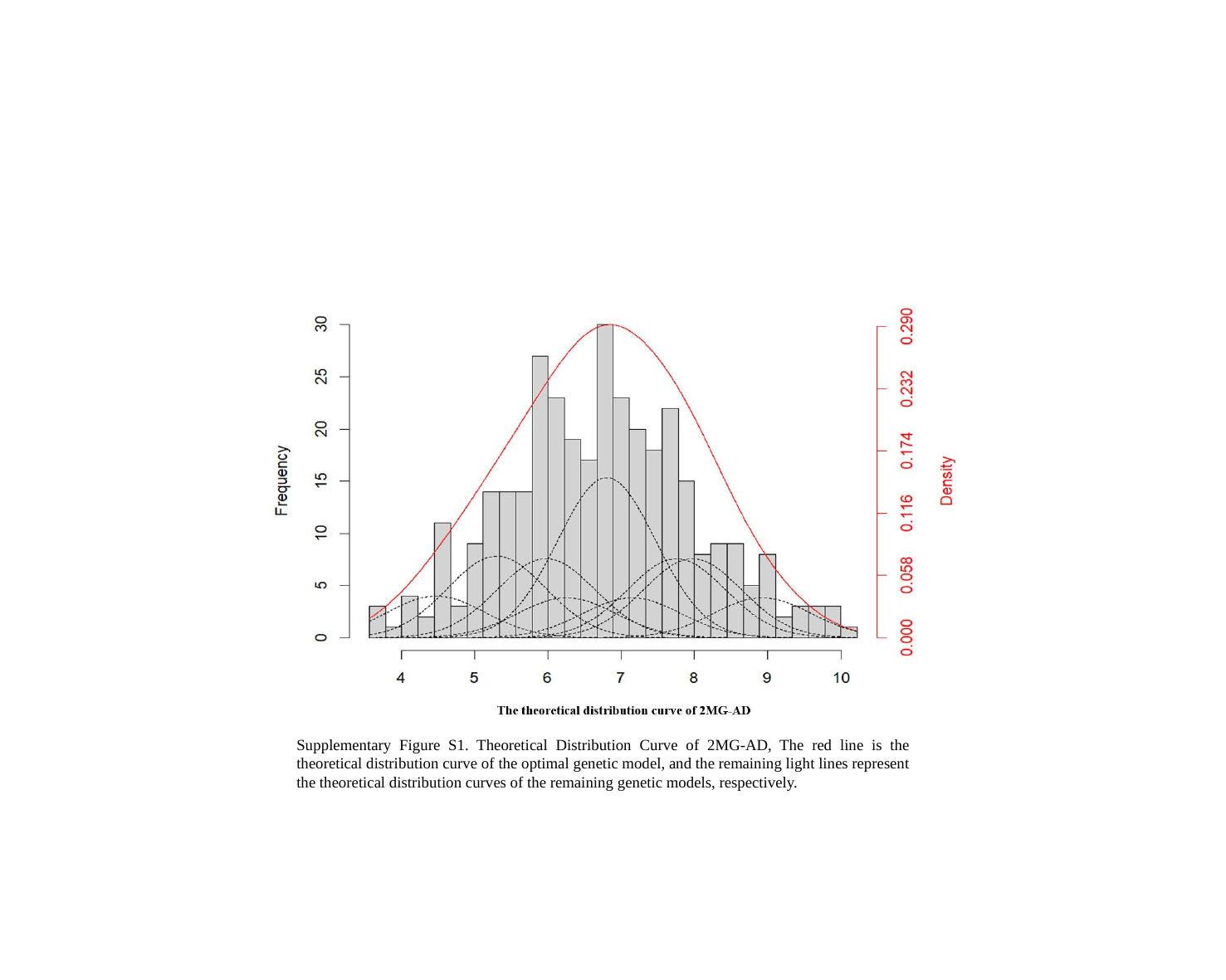

Supplementary Figure S1. Theoretical Distribution Curve of 2MG-AD, The red line is the theoretical distribution curve of the optimal genetic model, and the remaining light lines represent the theoretical distribution curves of the remaining genetic models, respectively.

## Slide 2
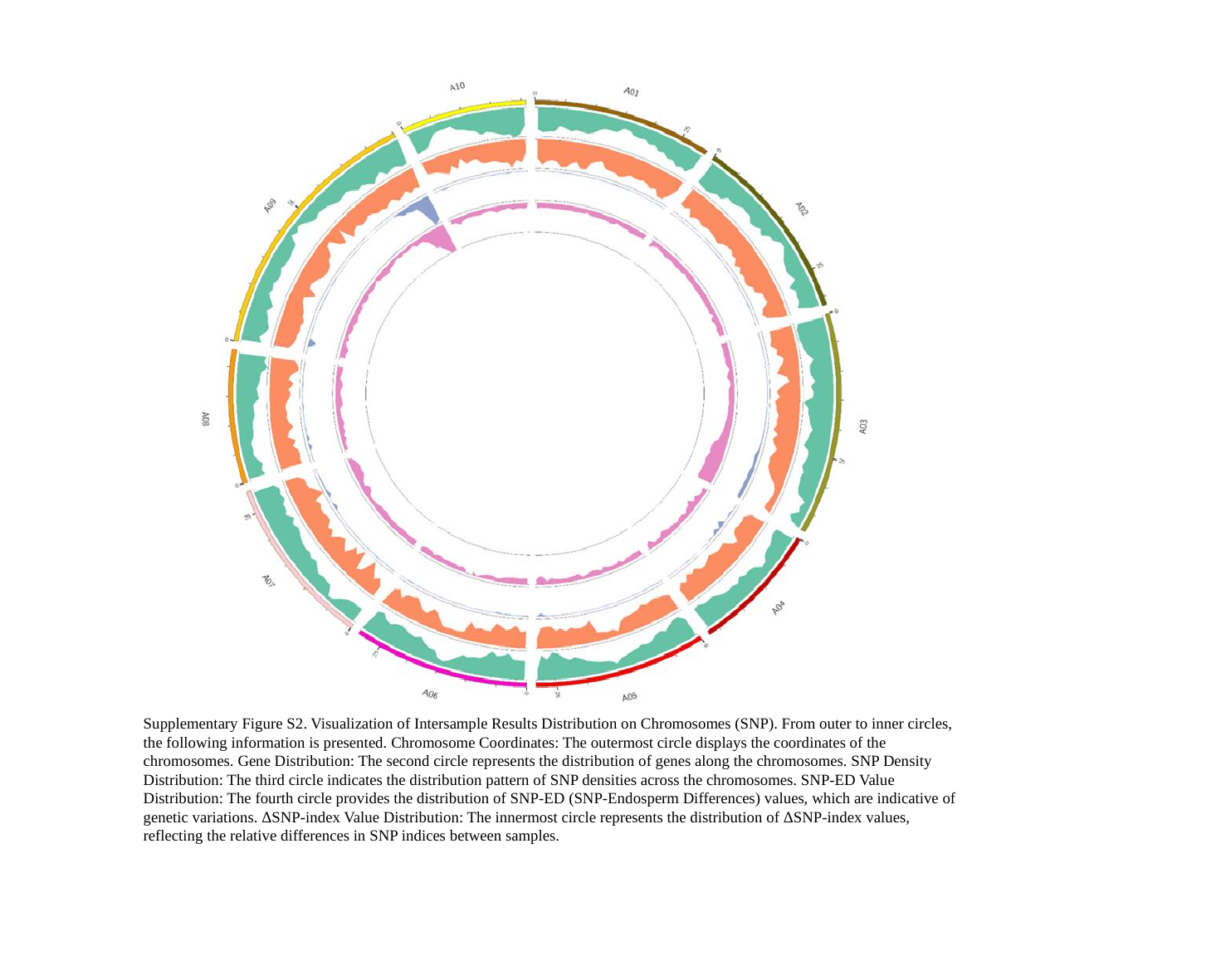

Supplementary Figure S2. Visualization of Intersample Results Distribution on Chromosomes (SNP). From outer to inner circles, the following information is presented. Chromosome Coordinates: The outermost circle displays the coordinates of the chromosomes. Gene Distribution: The second circle represents the distribution of genes along the chromosomes. SNP Density Distribution: The third circle indicates the distribution pattern of SNP densities across the chromosomes. SNP-ED Value Distribution: The fourth circle provides the distribution of SNP-ED (SNP-Endosperm Differences) values, which are indicative of genetic variations. ΔSNP-index Value Distribution: The innermost circle represents the distribution of ΔSNP-index values, reflecting the relative differences in SNP indices between samples.

## Slide 3
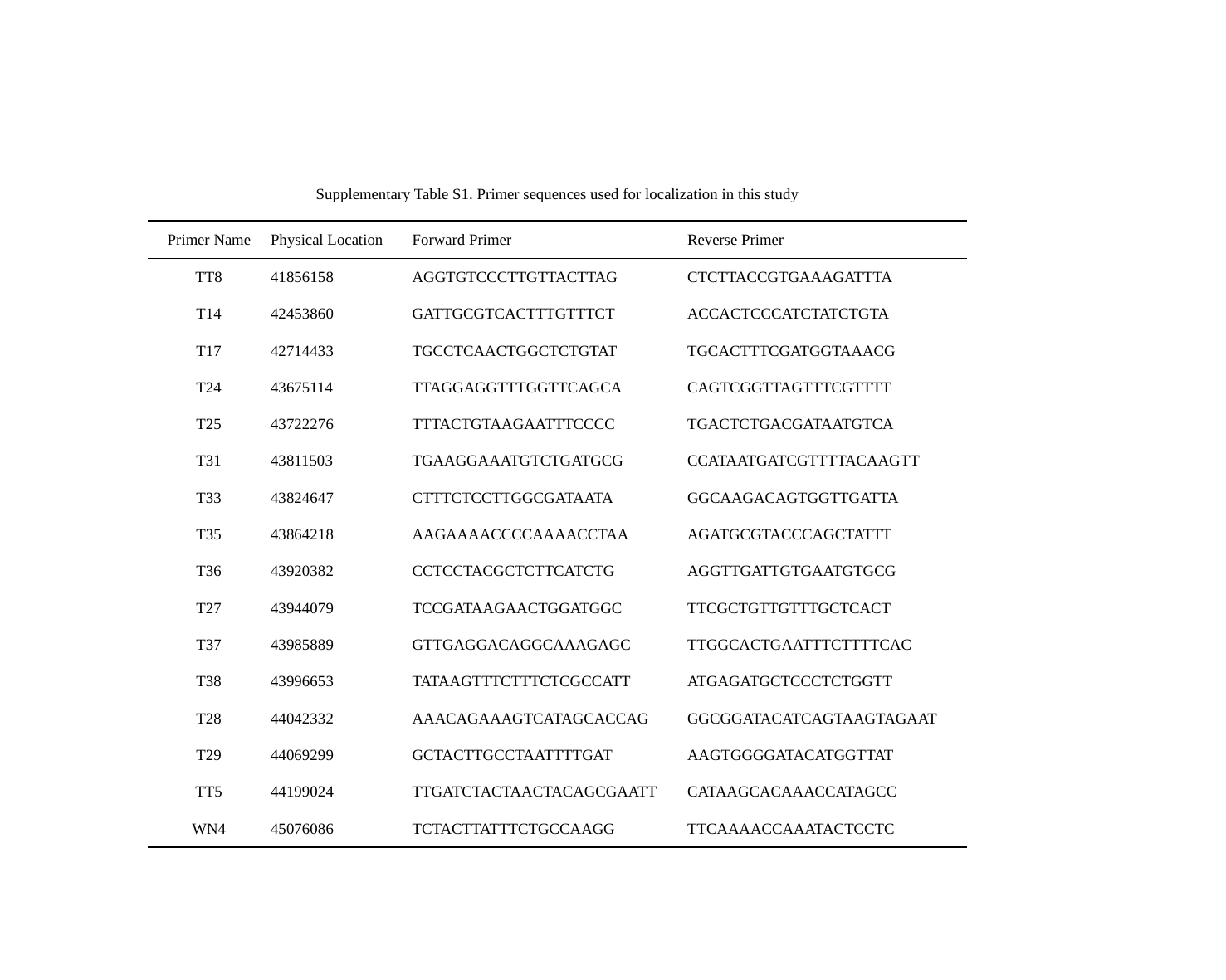

Supplementary Table S1. Primer sequences used for localization in this study
| Primer Name | Physical Location | Forward Primer | Reverse Primer |
| --- | --- | --- | --- |
| TT8 | 41856158 | AGGTGTCCCTTGTTACTTAG | CTCTTACCGTGAAAGATTTA |
| T14 | 42453860 | GATTGCGTCACTTTGTTTCT | ACCACTCCCATCTATCTGTA |
| T17 | 42714433 | TGCCTCAACTGGCTCTGTAT | TGCACTTTCGATGGTAAACG |
| T24 | 43675114 | TTAGGAGGTTTGGTTCAGCA | CAGTCGGTTAGTTTCGTTTT |
| T25 | 43722276 | TTTACTGTAAGAATTTCCCC | TGACTCTGACGATAATGTCA |
| T31 | 43811503 | TGAAGGAAATGTCTGATGCG | CCATAATGATCGTTTTACAAGTT |
| T33 | 43824647 | CTTTCTCCTTGGCGATAATA | GGCAAGACAGTGGTTGATTA |
| T35 | 43864218 | AAGAAAACCCCAAAACCTAA | AGATGCGTACCCAGCTATTT |
| T36 | 43920382 | CCTCCTACGCTCTTCATCTG | AGGTTGATTGTGAATGTGCG |
| T27 | 43944079 | TCCGATAAGAACTGGATGGC | TTCGCTGTTGTTTGCTCACT |
| T37 | 43985889 | GTTGAGGACAGGCAAAGAGC | TTGGCACTGAATTTCTTTTCAC |
| T38 | 43996653 | TATAAGTTTCTTTCTCGCCATT | ATGAGATGCTCCCTCTGGTT |
| T28 | 44042332 | AAACAGAAAGTCATAGCACCAG | GGCGGATACATCAGTAAGTAGAAT |
| T29 | 44069299 | GCTACTTGCCTAATTTTGAT | AAGTGGGGATACATGGTTAT |
| TT5 | 44199024 | TTGATCTACTAACTACAGCGAATT | CATAAGCACAAACCATAGCC |
| WN4 | 45076086 | TCTACTTATTTCTGCCAAGG | TTCAAAACCAAATACTCCTC |
